# Poly(ionic liquid)s Based Brush Type Nanomotor

**DOI:** 10.3390/mi9070364

**Published:** 2018-07-23

**Authors:** Yongjun Men, Yingfeng Tu, Wei Li, Fei Peng, Daniela A. Wilson

**Affiliations:** Institute for Molecules and Materials, Radboud University, Heyendaalseweg 135, 6525 AJ Nijmegen, The Netherlands; Y.Men@science.ru.nl (Y.M.); Y.Tu@science.ru.nl (Y.T.); w.li@student.ru.nl (W.L.); F.Peng@science.ru.nl (F.P.)

**Keywords:** nanomotor, brush, poly(ionic liquids), assembly assistant polymerization

## Abstract

A brush type nanomotor was fabricated via assembly assistant polymerization of poly(ionic liquid) and surface grafting polymerization. The method for large-scale fabrication of brush nanomotors with soft surfaces is described. These soft locomotive particles are based on core-shell brush nanoparticles assembled from poly(ionic liquid) as core and thermoresponsive PNIPAM as brush shells on which platinum nanoparticle (PtNP) were grown in situ. The particles show non-Brownian motion in H_2_O_2_ solution.

## 1. Introduction

The research on mimicking the autonomous motion in nature is a rapidly developing area lying at the nexus of chemistry, biology, medicine, environment and materials science [1,2,3,4,5,6,7,8,9,10,11,12,13,14,15,16,17,18,19,20,21]. Of crucial importance for this research is to design and fabricate suitable artificial micro/nanomotors for specific applications. To obtain asymmetric structures with various shapes at micro/nanometer scale, several approaches were employed to fabricate the nanomotors [7]. For example, micro-printing and metal sputtering methods were employed to prepare Janus nanospheres with partially coated catalysts that generate a product concentration gradient around the sphere, affording power to move the spheres along [22,23,24,25,26,27,28,29,30,31]. Templated electrodeposition method was commonly used to write sequences of metals into nanorods affording thus, nanomotors driven by self-electrophoresis, which is the movement in a self-induced electric field resulting from an asymmetrical distribution of ions [32,33,34,35,36,37,38,39]. Rolled-up nanotechnique and layer-by-layer assembly approach were also employed to prepare micro/nanotubes with inside grown or coated with catalysts, driving the nanomotors forward by bubbles propulsion [40,41,42,43,44]. Moreover, self-assembled nanodiscs with catalytic and magnetic nanoparticles on one side were also shown to be propelled via catalysis or guided magnetically due to the presence of the nanoparticles [45]. Our laboratory showed that self-assembly of amphiphilic block-copolymers and shape transformation is also a useful tool to fabricate stomatocyte nanomotors with entrapped catalysts in their stomachs. The movement of the soft assembled motors was induced by the fast bubble jet of oxygen generated by the catalyst inside the stomatocyte and it rapidly expelled through their narrow opening to pump up the motion of the nanomotors [46,47,48,49,50,51,52].

Each above-mentioned method exhibits its own advantages at certain conditions. But for application of micro/nanomotors in large amounts (tons) and at high concentrations still remains a challenge for these existing technologies. To solve this problem, the most challenging part is the asymmetrical anchoring of the catalyst on the carrier with non-equal distribution via a method suitable for amplification. Interestingly, research from the Yamamoto group demonstrated that even motors only composed of Pt metal clusters could also perform non-Brownian motion in H_2_O_2_ solution, and the motion was only dependent on the morphology of Pt particles [53]. This result indicates that colloidal methods can be employed to prepare nanomotors, as very recently demonstrated by the Pumera group [54]. Furthermore, a common feature of most reported motors is that they are mainly composed of metallic design with rigid and tough surfaces. This is in fact very different from that of most biological motors found in nature, which rely on soft interfaces and surfaces. As known, soft surfaces are easy to adapt to the surrounding environment in terms of their adjustable shapes and dynamic mobility, such as bacteria. Therefore, the design of active soft materials is of crucial importance for next generation artificial motors and promises to generate the multi-component soft machines of tomorrow. Beside their interesting properties, such as plasticity, unique shape, size, mechanical properties and possibility to respond to multiple stimuli, they also provide better interfaces with living systems.

Inspired from former reported studies [55,56,57], herein, we demonstrate a versatile colloid method for in situ growth of Pt nanoparticle (PtNP) as a catalyst on poly (*N*-isopropylacrylamide) (PNIPAM) brushes with non-equal distribution by controlling the concentration of Pt salt in a suitable range. This slight unequal distribution is shown to be sufficient to induce motion and therefore provide a solution for the large-scale fabrication of nanomotors with a soft interface in high concentration. Moreover, the movement of the nanomotor and the phase transition behaviour of the PNIPAM brush in different concentrations of H_2_O_2_ were investigated.

## 2. Materials and Methods 

### 2.1. Materials

1-Vinylimidazole (Aldrich, St. Louis, MO, USA, 99%), 11-bromo-1-undecanol (Aldrich, 98%), 2-bromo-2-methylpropionyl bromide (Aldrich, 98%), water-soluble nonionic azo initiator VA86 (Wako Chemicals, Tokyo, Japan), *N*,*N*,*N*′,*N*′,*N*″-pentamethyldiethylenetriamine (PMDETA; Aldrich, 99%), potassium bromide (Aldrich, 99.95%), copper(I) bromide (Aldrich, 99.99%), K_2_PtCl_4_ (Aldrich, 98%), NaBH_4_ (Aldrich, 99%) were used as received without further purifications. *N*-Isopropylacrylamide (NIPAM; Aldrich, 97%) was recrystallized three times from a mixture of toluene/*n*-hexane (*v*/*v*) 1:1 prior to use. All solvents used were of analytic grade.

### 2.2. Methods

#### 2.2.1. Synthesis of the Ionic Liquid Monomers (ILM)

The same as a former reported paper [58]. Synthesis of 3-(11-hydroxyundecanyl)-1-vinylimidazolium bromide (ILOH): 0.1 mol of 1-vinylimidazole, 0.1 mol of 11-bromo-1-undecanol, and 40 mL of methanol were loaded into a 100 mL flask. The mixture was stirred at 60 °C for 24 h. After cooling down, the reaction mixture was added dropwise into 1 L of diethyl ether. The white precipitate was filtered off, washed three times with diethyl ether, and then dried at 40 °C till constant weight (yield: 80%).

The synthesis of 3-[11-(2-bromo-2-methyl-1-oxopropoxy)undecyl]-1-vinylimidazolium bromide (ILM): 0.05 mol of ILOH, 0.1 mol K_2_CO_3_ was mixed with 100 mL dry dichloromethane in a 250 mL flask. The mixture was stirred in an ice bath for 15 min, and then 0.1 mol 2-bromoisobutyryl bromide in 50 mL dry dichloromethane was added dropwise into the solution. The whole process was protected by argon. The reaction processed for 12 h. The mixture was filtered to remove KBr and unreacted K_2_CO_3_. The filtrate was concentrated by rotary evaporation until about 50 mL was left, and then it was added dropwise into 1L of diethyl ether. Viscous light yellow liquid was precipitated at the bottom, washed three times with diethyl ether, and then dried at 40 °C till constant weight (yield: 76%). The ^1^H-NMR were shown in Figure S1.

#### 2.2.2. Preparation of Poly(ionic liquid) Nanoparticle (NP-Br)

In a typical reaction, polymerization was carried out in a 100 mL Schlenk glass. 50 mL of water containing 2.5 g of ILM and 0.025 g of VA86 was first introduced into the reactor, and then deoxygenated by three cycles of freeze-pump-thaw procedure and was backfilled with argon. The flask was then stirred in an oil bath thermostated at 70 °C for 16 h. The formed dispersion was then cooled down to room temperature. The reaction mixture was dialyzed against Milli-Q water (molecular weight cut off at 12,000–14,000 g/mol) for 5 days (being changed 4 times per day) to remove unreacted monomer and initiator.

#### 2.2.3. Grafting PNIPAM onto Poly(ionic liquid) Nanoparticle (NP*-g-*PNIPAM) by ATRP

A suspension of NP-Br (30 mL, *d* = 69 nm, 2.5 wt %) carrying the ATRP initiator layer was degassed for 2.5 h by argon continuous bubbling. NIPAM (1.697 g, 15 mmol), PMTETA (52.0 mg, 300 μmol), and CuBr (43 mg, 300 μmol) were added into NP-Br suspension under stirring at room temperature (21 °C) in the glove box. All reactions were stopped in 30 min due to an extremely high reaction rate. The reaction mixture was purified though dialysis membranes with a molecular weight cutoff of 12,000–14,000 g/mol. Cu remained 310 ppb (measured by EA).

#### 2.2.4. In Situ Grown PtNP on PNIPAM Brushes

PtNP were prepared in the presence of NP-*g*-PNIPAM particles. In a typical experiment, 5 mL NP-*g*-PNIPAM particles solution (solid content 4.91 wt %), 0.05 mL of 0.02 M K_2_PtCl_6_ was added to a glass test tube with gentle stirring. After that, 0.5 mL of an aqueous 0.01 M ice-cold NaBH_4_ solution was added at once, followed by rapid magnetic stirring for 30 min.

All characterization methods are put in the supporting information.

## 3. Results and Discussion

### 3.1. Brush Nanomotor Fabricaiton

Brush nanoparticles with poly(ionic liquid) (PILs) as core and PNIPAM as polymer shell were synthesized according to a procedure described by Yuan et al. [58]. PILs, a new type of polyelectrolyte composed of ionic liquid units, were chosen to fabricate nanolatex as core due to their high stability and facile fabrication method as well as high charge density [59,60,61,62].

**Scheme 1:**
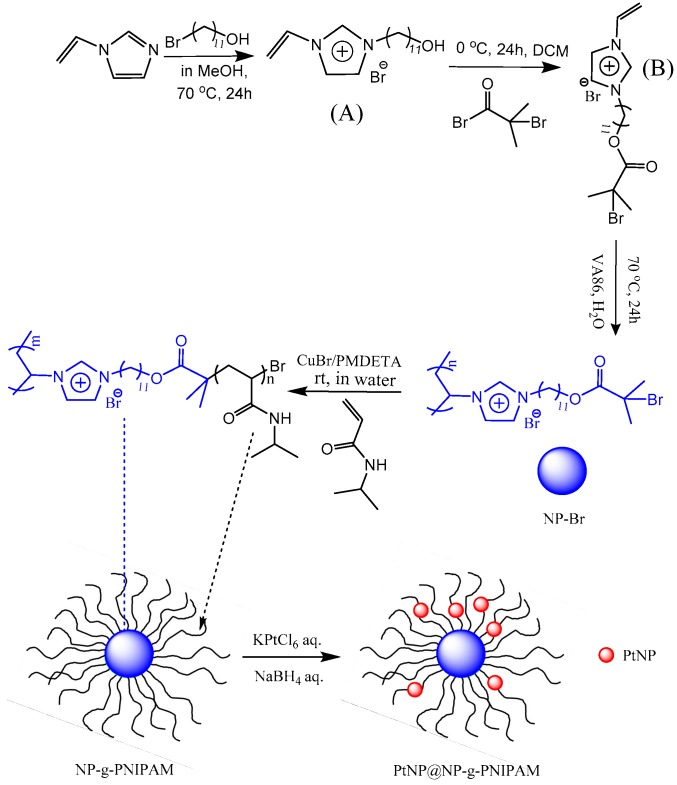
Synthetic route to brush nanomotor with a PIL core and a PtNP@PNIPAM shell. Compound A is hydroxyl-terminated IL monomer; compound B is bromide-terminated IL monomer; the blue nanoparticle presents polymerized IL nanoparticles as a core; PNIPAM brushes growing from NP are presented as dark lines; the red sphere is on behalf of PtNP.

**Figure 1:**
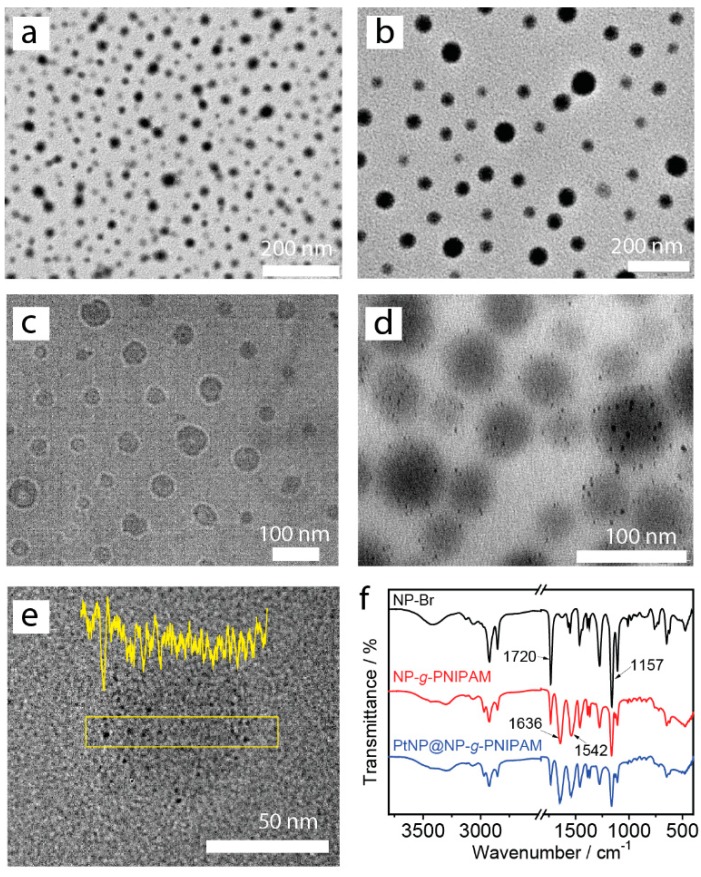
TEM images of NP-Br (**a**), NP-PNIPAM (**b**), PtNP@NP-*g*-PNIPAM (**d**,**e**), cryo-TEM image of Pt NP@NP-PNIPAM (**c**), FTIR spectra of NP-Br, NP-*g*-PNIPAM, and PtNP@NP-*g*-PNIPAM (**f**), and the intensity distribution of the selected area in (**e**).

As shown in Scheme 1, an ionic liquid monomer (ILM) bearing a distal ATRP initiation group in the alkyl tail (compound B) was synthesized via a quaternization reaction of hydroxyl-terminated IL (compound A) with 11-bromo-1-undecanol at 0 °C in dichloromethane. The structure of the ILM was confirmed via proton nuclear magnetic resonance (^1^H-NMR) spectrum. The signal of the six protons (>C(CH_3_)_2_) from α-bromoisobutyroyl overlapped with the two methylene protons in >N-CH_2_-CH_2_- at 1.9 ppm (Figure S1), with about a four times larger integral area than that of the two protons adjacent to the imidazolium cation, indicating a good quantitative conversion. The poly(ionic liquid) nanoparticle (NP-Br) was prepared with a monomer concentration of 5 wt % at 70 °C, initiated by a non-ionic initiator VA86 (3 wt % with regard to the ILM). The obtained particle size was around 69 nm from DLS results (Figure S2), and its zeta potential was +43 (Figure S3) due to the high charge density on the surface of PIL latex. The transmission electron microscopy (TEM) image (Figure 1a) displays well-dispersed particle morphology, showing a similar particle size as measured by DLS. PNIPAM was grafted on to the PIL latex (2.5 wt %) via ATRP in aqueous solution using CuBr as catalyst and *N*,*N*,*N*′,*N*′,*N*″-pentamethyldiethylenetriamine (PMDETA) as ligand. The [CuBr]:[PMDETA]:[PMDETA] molar ratio was set to 1:1:50. After 30 min of reaction, the mixture was purified via dialysis for 7 days with frequently changing water to remove the residues, including the catalyst, ligand and unreacted monomer. To test the residue concentration of Cu after dialysis, inductively coupled plasma atomic emission spectroscopy (ICP-AES) was employed for a quantitative measurement. The result shows that about 310.85 ppb of Cu still existed in the suspension, which is trace amount due to the strong coordination between copper and PNIPAM brush. The size of the grafted nanoparticle (NP*-g-*PNIPAM) was controlled to 182 nm via selecting an optimized reaction time and monomer ratio, as measured by DLS (Figure S3).

The size shown from the TEM image (Figure 1b) is much smaller than 182 nm due to the drying effect, but still larger than the size of the NP-Br. The zeta potential of NP*-g-*PNIPAM particles (Figure S2) is an order of magnitude lower than that of NP-Br, which was expected due to the higher relative hydrophilicity of the grafted particles. In this case, the PNIPAM is more hydrophilic and forms a shell in order to stabilize the particle via steric interaction and consequently drastically reduces the zeta potential compared with the NP-Br [63]. The functionalization of the particles with the thermoresponsive brushes was further analyzed by FTIR spectroscopy (Figure 1f). The characteristic peaks from the PNIPAM segment at 1636 and 1542 cm^−1^ (primary amide C=O, N-H strech) and the peaks of the PIL segment at 1720 (C=O stretch) and 1157 cm^−1^ (C-H stretch of imidazole ring) were all presented within the spectrum of NP*-g-*PNIPAM, demonstrating the successful grafting from the PIL nanoparticles of the PNIPAM.

PtNP was grown on the PNIPAM brushes in situ according to the method reported by Lu [55,64]. PtNP with an average diameter of ~5 nm was prepared via the reduction of K_2_PtCl_4_ with NaBH_4_, and immobilized onto the PNIPAM brushes. According to the literature [65], the mechanism of particle formation prospects that nitrogen in PNIPAM coordinates with PtCl_4_^2−^, which decreases the potential of Pt/PtCl_4_^2−^ and promotes the reduction of PtCl_4_^2−^. Cryo-TEM image (Figure 1c) of the grafted particles showed the relatively spherical morphology and high dispersity of the grafted PtNP@NP-PNIPAM nanoparticles. Very interestingly, the TEM image (Figure 1d,e) showed that PtNP was slightly non-equally distributed on the brushes, as demonstrated by the intensity distribution of the selected area, which is larger for metal particles such as PtNP. As shown in Figure 1e, the intensity of the left side of the particle is stronger than the right side, suggesting larger distribution of PtNP on the left side of the grafted particle. This structure may have provided the opportunity to generate an asymmetric brush structure able to propel the motor in H_2_O_2_ fuel. One suggested reason for this asymmetrical distribution might be the non-equal changes of aggregation with the formed intermediate PtNP. No secondary Pt nanoparticles could be found outside the template particles, most probably due to the strong complexation of the metalate ions with the nitrogen atoms of the PNIPAM brushes and the relatively small amount of the Pt precursor. Additionally, no peak appeared between 1–10 nm in the DLS (Figure S3) plot of the brush nanomotor solution. Therefore, no further purification of the as-synthesized nanoparticles was required, showing a facile way to prepare this non-equally distributed Pt@NP*-g-*PNIPAM particle towards large-scale. Another reasonable explanation for the non-Brownian movement of the particles is the possible generation of asymmetric shape of the nanomotor during catalysis due to the known exothermal effect during decomposition of the fuel and the further effect of the fuel on the brush.

**Figure 2:**
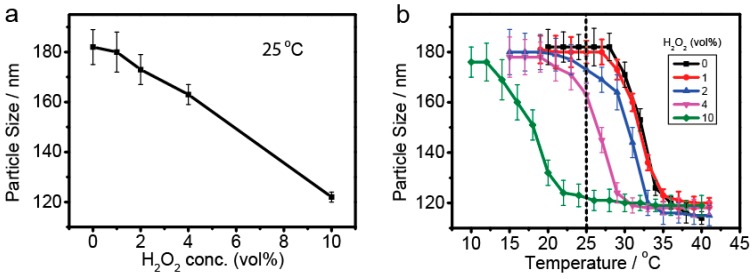
(**a**) The particle size of the PtNP-free brush particles in various H_2_O_2_ concentrations as measured by DLS at 25 °C. (**b**) Size variation of the PtNP-free brush particles versus temperature in various H_2_O_2_ concentrations by DLS measurement.

As known from literature, the lower critical solution temperature (LCST) of PNIPAM is dependent on the H_2_O_2_ concentrations [66]. To understand the brush state of our nanomotor system, we measured the change in the size of the brush nanoparticle without Pt catalyst in different H_2_O_2_ concentrations via DLS. The results showed that the particle sizes decreased from ~182 nm to ~120 nm with raising the temperature at various H_2_O_2_ concentrations (Figure 2b). When the H_2_O_2_ concentration was as low as 1 vol %, the size changed only slightly compared with that in water. However, by increasing the H_2_O_2_ concentrations from 2 to 10 vol %, the starting shrinking temperature decreased from 21 to 10 °C. The reason might be that two hydroxide groups in H_2_O_2_ can be both hydrogen-bond accepters and donators, allowing the formation of cycle-shaped structure with water molecules other than those hydrated with PNIPAM polymer [67]. When comparing the half shrinking state of the particle at 0, 1, 2, 4 and 10 H_2_O_2_ vol %, we observed a decrease in the diameter by 30 nm for the corresponding LCST temperatures at 32, 32, 31, 26 and 18 °C. Since the movement analysis was carried out at 25 °C, we plotted the particle size as function of H_2_O_2_ concentration at this temperature for a direct view (Figure 2a). We observed an almost linear decrease of the particle size with the increase in the fuel concentration, while for concentrations below 1 vol % the size of the particles shrunk by only <2 nm. These results show that the PNIPAM brushes have almost no influence on the particle size as well motion, if the fuel concentration is kept below 1 vol %. At high H_2_O_2_ concentration, however, complex factors that can affect the motion of the particles as well the mechanism of motion have to be considered. Firstly, high H_2_O_2_ concentration will shrink the PNIPAM brushes and increase the polymer and catalyst density around the nanoparticle as well as its hydrophobicity, which would decrease the catalytic efficiency of PtNP. Secondly, hot spots might be generated around the PtNPs due to the exothermic reaction of the decomposition of hydrogen peroxide, leading to a phase separation around high concentrated PtNP places. Thirdly, air-water interface, generated from oxygen bubble, might have a significant effect on the motion of the nanosized motor.

### 3.2. Motion of Brush Type Nanomotor

Therefore, to simplify the experimental conditions, in this work we only focused on investigating the motion with an H_2_O_2_ concentration lower than 0.5 vol %. A new technique, Nanoparticle Tracking Analysis (NTA), which has been demonstrated in our former studies, was employed to prove the expected directional movement of the brush nanomotor [46,48]. NTA technique was used to track the non-Brownian motion that arises from the fast movement of the non-equally distributed Pt on NP*-g-*PNIPAM brush nanoparticles in the presence of the H_2_O_2_ fuel.

**Figure 3:**
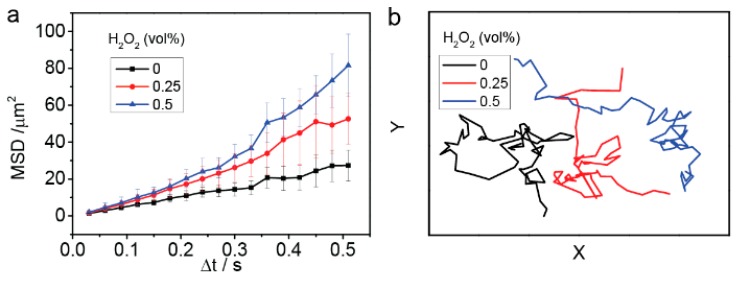
(**a**) Average mean square displacement (MSD) of the brush nanomotor in different concentrations of H_2_O_2_ calculated from the tracking coordinates of 90 particles from the major size distribution. (**b**) Typical trajectories of the supramolecular nanomotor at 0, 0.25 and 0.5 vol % of H_2_O_2_.

Indeed, small amounts of fuel (H_2_O_2_, 0.25 vol %) showed an increase in the mean square displacement (MSD) when compared to the Brownian motion typically observed in the absence of fuel and a linear fit for the MSD (Figure 3a). When the concentration of H_2_O_2_ was raised to 0.5 vol %, the structures showed a propulsive and a parabolic fit of MSD. The trajectory of the brush nanomotor with and without the hydrogen peroxide fuel presented the same behaviour (Figure 3b). To give further demonstration of the non-Brownian motion of the particles, directionality and the length of center of mass were measured [68]. The directness is calculated by comparing the euclidian distance to the accumulated distance. The values of directness are between 0 and 1, and the higher the value indicates the larger the non-Brownian motion. The center of mass represents the spatial averaged point of all particle endpoints. It is only one point, and the coordinates can be either positive or negative. The difference in the center of mass at the beginning and end of the experiment is called the length of the center of mass. Particles performing non-Brownian motion have longer length of the center of mass. Table 1 shows the directionalities of the Pt@NP*-g-*PNIPAM nanoparticles increase from 0.16 to 0.45 when the concentration of hydrogen peroxide rises from 0 to 0.5 vol %. These results indicate that without fuel, the brush nanomotor followed the typical random walk of a Brownian motion and did not show directionality, and the length of center of mass was very small, only 1.22 μm in 1 s. While in the presence of 0.5 vol % H_2_O_2_ fuel, the directionality is as high as 0.45, indicating a non-Brownian motion, and the length of center of mass increases to 6.59, much larger than the data measured in pure water. At a lower fuel concentration (H_2_O_2_, 0.25 vol %), the same behaviour was observed, the motors presented a much lower directionality and length of center of mass, demonstrating that the movement of the nanomotors is dependent on the fuel concentration. The suggested motion mechanism of this PNIPAM brush nanomotors is nano-bubbles propulsion. The oxygen generated on the catalyst surface dissolved in the solution around the nanoparticles, which continued to diffuse into the bubble created at some rough point of the particle, causing it to grow until it reached the detachment radius and was released from the surface; a momentum change resulted from the detachment and induced a driving force away from the catalyst surface [69]. In this brush nanomotor system, oxygen bubbles generated from different spots of the particles due to the non-equally distributed PtNPs on the brushes, resulting in a slower speed of motion compared to normal Janus motors.

## 4. Conclusions

In summary, a brush nanomotor was successfully produced with a colloid method by in situ growing PtNP on the spherical PNIPAM brush particles bearing a PIL nanoparticle core. The PtNP was non-equally distributed on the brush sphere, inducing a non-Brownian motion in H_2_O_2_ fuel. PNIPAM brush was demonstrated to have little influence on the motion of the nanomotor when H_2_O_2_ concentration was lower than 0.5 vol %. Future research will focus on extending this method to other nanosystems, such as nanogel and self-assembled structures, which will be helpful to entrap and release drugs for biomedical application. Moreover, the highly charged PIL brush nanomotor system might be used in active charge transporter applications.

## Figures and Tables

**Table 1:** Data for the brush nanomotor in pure water and hydrogen peroxide with different concentrations.

H_2_O_2_ (wt %)	Directionality (1 s) ^a^	Center of Mass (Length, μm) ^a^
0	0.16	1.22
0.25	0.25	2.19
0.50	0.45	6.59

^a^ Data were obtained by tracking of 15 nanoparticles for each experiment.

## Supplementary Materials

The following are available online at http://www.mdpi.com/2072-666X/9/7/364/s1, Figure S1: 1H-NMR spectra of 3-(11-hydroxyundecanyl)-1-vinylimidzalium bromide before (black line) and after (red line) esterification reaction with α-bromoisobutyryl bromide. Figure S2. Zeta-Potential values of NP-Br, NP*-g-*PNIPAM and PtNP@ NP*-g-*PNIPAM. Figure S3 Size of NP-Br, NP*-g-*PNIPAM and PtNP@ NP*-g-*PNIPAM measured by DLS.

## References

[1] Ismagilov R.F., Schwartz A., Bowden N., Whitesides G.M. (2002). Autonomous movement and self-assembly. Angew. Chem. Int. Ed..

[2] Feringa B.L. (2001). In control of motion: From molecular switches to molecular motors. Acc. Chem. Res..

[3] Besenbacher F., Norskov J.K. (2000). How to power a nanomotor. Science.

[4] Schliwa M., Woehlke G. (2003). Molecular motors: Switching on kinesin. Nature.

[5] Chen Y., Wang M., Mao C. (2004). An autonomous DNA nanomotor powered by a DNA enzyme. Angew. Chem. Int. Ed..

[6] Paxton W.F., Sundararajan S., Mallouk T.E., Sen A. (2006). Chemical locomotion. Angew. Chem. Int. Ed..

[7] Wang H., Pumera M. (2015). Fabrication of micro/nanoscale motors. Chem. Rev..

[8] Men Y., Peng F., Wilson D.A. (2016). Micro/nanomotors via self-assembly. Sci. Lett. J..

[9] Maria G., Weiz S.M., Schmidt O.G., Mariana-Sánchez M. (2018). Self-propelled micro/nanoparticle motors. Part. Part. Syst. Charact..

[10] Mariana-Sánchez M., Magdanz V., Guix M., Fomin V.M., Schmidt O.G. (2018). Swimming microrobots: Soft, reconfigurable, and smart. Adv. Funct. Mater..

[11] Kim K., Guo J., Liang Z.X., Zhu F.Q., Fan D.L. (2016). Man-made rotary nanomotors: A review of recent developments. Nanoscale.

[12] Choi H., Lee G.-H., Kim K.S., Hahn S.K. (2018). Light-guided nanomotor systems for autonomous photothermal cancer therapy. ACS Appl. Mater. Interfaces.

[13] Xuan M., Mestre R., Gao C., Zhou C., He Q., Sanchez S. (2018). Noncontinuous super-diffusive dynamics of a light-activated nanobottle motor. Angew. Chem. Int. Ed..

[14] Santiago I. (2018). Nanoscale active matter matters: Challenges and opportunities for self-propelled nanomotors. Nano Today.

[15] Li M., Zhang H., Liu M., Dong B. (2018). A light-powered shape-configurable micromachine. Mater. Horiz..

[16] Men Y., Li W., Janssen G.-J., Rikken R.S., Wilson D.A. (2018). Stomatocyte in stomatocyte: A new shape of polymersome induced via chemical-addition methodology. Nano Lett..

[17] Zarei M., Zarei M. (2018). Self-propelled micro/nanomotors for sensing and environmental remediation. Small.

[18] Jiao J., Xu D., Liu Y., Zhao W., Zhang J., Zheng T., Feng H., Ma X. (2018). Mini-emulsionfabricated magnetic and fluorescent hybrid Janus micro-motors. Micromachines.

[19] Lin Z., Gao C., Chen M., Lin X., He Q. (2018). Collective motion and dynamic self-assembly of colloid motors. Curr. Opin. Colloid Interface Sci..

[20] Safdar M., Khan S.U., Jänis J. (2018). Progress toward catalytic micro-and nanomotors for biomedical and environmental applications. Adv. Mater..

[21] Luo M., Feng Y., Wang T., Guan J. (2018). Micro-/nanorobots at work in active drug delivery. Adv. Funct. Mater..

[22] Howse J.R., Jones R.A.L., Ryan A.J., Gough T., Vafabakhsh R., Golestanian R. (2007). Self-motile colloidal particles: From directed propulsion to random walk. Phys. Rev. Lett..

[23] Guell O., Sagues F., Tierno P. (2011). Magnetically driven Janus micro-ellipsoids realized via asymmetric gathering of the magnetic charge. Adv. Mater..

[24] Wu Y., Wu Z., Lin X., He Q., Li J. (2012). Autonomous movement of controllable assembled Janus capsule motors. ACS Nano.

[25] Gao W., D’Agostino M., Garcia-Gradilla V., Orozco J., Wang J. (2012). Multi-fuel driven Janus micromotors. Small.

[26] Mou F., Chen C., Zhong Q., Yin Y., Ma H., Guan J. (2014). Autonomous motion and temperature-controlled drug delivery of Mg/Pt-poly(*N*-isopropylacrylamide) Janus micromotors driven by simulated body fluid and blood plasma. ACS Appl. Mater. Interfaces.

[27] Ma X., Sanchez S. (2015). A bio-catalytically driven Janus mesoporous silica cluster motor with magnetic guidance. Chem. Commun..

[28] Wu Y., Si T., Lin X., He Q. (2015). Near infrared-modulated propulsion of catalytic Janus polymer multilayer capsule motors. Chem. Commun..

[29] Wu Y., Lin X., Wu Z., Möhwald H., He Q. (2014). Self-propelled polymer multilayer Janus capsules for effective drug delivery and light-triggered release. ACS Appl. Mater. Interfaces.

[30] Wu Z., Lin X., Zou X., Sun J., He Q. (2015). Biodegradable protein-based rockets for drug transportation and light-triggered release. ACS Appl. Mater. Interfaces.

[31] Xuan M.J., Shao J.X., Lin X.K., Dai L., He Q. (2014). Self-propelled Janus mesoporous silica nanomotors with sub-100 nm diameters for drug encapsulation and delivery. Chemphyschem.

[32] Wang Y., Hernandez R.M., Bartlett D.J., Bingham J.M., Kline T.R., Sen A., Mallouk T.E. (2006). Bipolar electrochemical mechanism for the propulsion of catalytic nanomotors in hydrogen peroxide solutions. Langmuir.

[33] He Y., Wu J., Zhao Y. (2007). Designing catalytic nanomotors by dynamic shadowing growth. Nano Lett..

[34] Demirok U.K., Laocharoensuk R., Manesh K.M., Wang J. (2008). Ultrafast catalytic alloy nanomotors. Angew. Chem. Int. Ed..

[35] Laocharoensuk R., Burdick J., Wang J. (2008). Carbon-nanotube-induced acceleration of catalytic nanomotors. ACS Nano.

[36] Calvo-Marzal P., Sattayasamitsathit S., Balasubramanian S., Windmiller J.R., Dao C., Wang J. (2010). Propulsion of nanowire diodes. Chem. Commun..

[37] Gao W., Sattayasamitsathit S., Manesh K.M., Weihs D., Wang J. (2010). Magnetically powered flexible metal nanowire motors. J. Am. Chem. Soc..

[38] Kagan D., Laocharoensuk R., Zimmerman M., Clawson C., Balasubramanian S., Kong D., Bishop D., Sattayasamitsathit S., Zhang L., Wang J. (2010). Rapid delivery of drug carriers propelled and navigated by catalytic nanoshuttles. Small.

[39] Manesh K.M., Cardona M., Yuan R., Clark M., Kagan D., Balasubramanian S., Wang J. (2010). Template-assisted fabrication of salt-independent catalytic tubular microengines. ACS Nano.

[40] Mei Y., Huang G., Solovev A.A., Ureña E.B., Mönch I., Ding F., Reindl T., Fu R.K.Y., Chu P.K., Schmidt O.G. (2008). Versatile approach for integrative and functionalized tubes by strain engineering of nanomembranes on polymers. Adv. Mater..

[41] Solovev A.A., Mei Y., Urena E.B., Huang G., Schmidt O.G. (2009). Catalytic microtubular jet engines self-propelled by accumulated gas bubbles. Small.

[42] Sanchez S., Solovev A.A., Mei Y.F., Schmidt O.G. (2010). Dynamics of biocatalytic microengines mediated by variable friction control. J. Am. Chem. Soc..

[43] Solovev A.A., Sanchez S., Pumera M., Mei Y.F., Schmidt O.G. (2010). Magnetic control of tubular catalytic microbots for the transport, assembly, and delivery of micro-objects. Adv. Funct. Mater..

[44] Wu Z., Wu Y., He W., Lin X., Sun J., He Q. (2013). Self-propelled polymer-based multilayer nanorockets for transportation and drug release. Angew. Chem. Int. Ed..

[45] Dong B., Zhou T., Zhang H., Li C.Y. (2013). Directed self-assembly of nanoparticles for nanomotors. ACS Nano.

[46] Wilson D.A., Nolte R.J.M., van Hest J.C.M. (2012). Autonomous movement of platinum-loaded stomatocytes. Nat. Chem..

[47] Wilson D.A., Nolte R.J.M., van Hest J.C.M. (2012). Entrapment of metal nanoparticles in polymer stomatocytes. J. Am. Chem. Soc..

[48] Wilson D.A., de Nijs B., van Blaaderen A., Nolte R.J., van Hest J.C.M. (2013). Fuel concentration dependent movement of supramolecular catalytic nanomotors. Nanoscale.

[49] Abdelmohsen L.K.E.A., Peng F., Tu Y., Wilson D.A. (2014). Micro- and nano-motors for biomedical applications. J. Mater. Chem. B.

[50] Rikken R.S.M., Kerkenaar H.H.M., Nolte R.J.M., Maan J.C., van Hest J.C.M., Christianen P.C.M., Wilson D.A. (2014). Probing morphological changes in polymersomes with magnetic birefringence. Chem. Commun..

[51] Kim K.T., Zhu J., Meeuwissen S.A., Cornelissen J.J.L.M., Pochan D.J., Nolte R.J.M., van Hest J.C.M. (2010). Polymersome stomatocytes: Controlled shape transformation in polymer vesicles. J. Am. Chem. Soc..

[52] Meeuwissen S.A., Kim K.T., Chen Y., Pochan D.J., van Hest J.C.M. (2011). Controlled shape transformation of polymersome stomatocytes. Angew. Chem. Int. Ed..

[53] Yamamoto D., Mukai A., Okita N., Yoshikawa K., Shioi A. (2013). Catalytic micromotor generating self-propelled regular motion through random fluctuation. J. Chem. Phys..

[54] Teo W.Z., Zboril R., Medrik I., Pumera M. (2016). Fe^0^ nanomotors in ton quantities (10^20^ units) for environmental remediation. Chem. Eur. J..

[55] Lu Y., Yuan J., Polzer F., Drechsler M., Preussner J. (2010). In situ growth of catalytic active Au−Pt bimetallic nanorods in thermoresponsive core−shell microgels. ACS Nano.

[56] Wunder S., Polzer F., Lu Y., Mei Y., Ballauff M. (2010). Kinetic analysis of catalytic reduction of 4-nitrophenol by metallic nanoparticles immobilized in spherical polyelectrolyte brushes. J. Phys. Chem. C.

[57] Chen K., Zhu Y., Zhang Y., Li L., Lu Y., Guo X. (2011). Synthesis of magnetic spherical polyelectrolyte brushes. Macromolecules.

[58] Men Y., Drechsler M., Yuan J. (2013). Double-stimuli-responsive spherical polymer brushes with a poly(ionic liquid) core and a thermoresponsive shell. Macromol. Rapid Commun..

[59] Men Y., Schlaad H., Yuan J. (2013). Cationic poly(ionic liquid) with tunable lower critical solution temperature-type phase transition. ACS Macro Lett..

[60] Men Y., Kuzmicz D., Yuan J. (2014). Poly(ionic liquid) colloidal particles. Curr. Opin. Colloid Interface Sci..

[61] Men Y., Siebenburger M., Qiu X., Antonietti M., Yuan J. (2013). Low fractions of ionic liquid or poly(ionic liquid) can activate polysaccharide biomass into shaped, flexible and fire-retardant porous carbons. J. Mater. Chem. A.

[62] Yuan J., Mecerreyes D., Antonietti M. (2013). Poly(ionic liquid)s: An update. Prog. Polym. Sci..

[63] Yu R., Tauer K. (2014). From particles to stabilizing blocks—Polymerized ionic liquids in aqueous heterophase polymerization. Polym. Chem..

[64] Mei Y., Lu Y., Polzer F., Ballauff M., Drechsler M. (2007). Catalytic activity of palladium nanoparticles encapsulated in spherical polyelectrolyte brushes and core−shell microgels. Chem. Mater..

[65] Frattini A., Pellegri N., Nicastro D., de Sanctis O. (2005). Effect of amine groups in the synthesis of Ag nanoparticles using aminosilanes. Mater. Chem. Phys..

[66] Liu L., Wang T., Liu C., Lin K., Ding Y., Liu G., Zhang G. (2013). Mechanistic insights into amplification of specific ion effect in water–nonaqueous solvent mixtures. J. Phys. Chem. B.

[67] Kulkarni A.D., Pathak R.K., Bartolotti L.J. (2005). Structures, energetics, and vibrational spectra of H_2_O_2_···(H_2_O)_n_, n = 1−6 clusters:  Ab initio quantum chemical investigations. J. Phys. Chem. A.

[68] Kline T.R., Paxton W.F., Mallouk T.E., Sen A. (2005). Catalytic nanomotors: Remote-controlled autonomous movement of striped metallic nanorods. Angew. Chem. Int. Ed..

[69] Gibbs J.G., Zhao Y.-P. (2009). Autonomously motile catalytic nanomotors by bubble propulsion. Appl. Phys. Lett..

