# Supplementary material for: Poly(ionic liquid)s Based Brush Type Nanomotor
Source: Micromachines (Basel). 2018 Jul 23;9(7):364. doi: 10.3390/mi9070364 (PMC6082249; doi:10.3390/mi9070364)
Supplement: Supplementary file 1 [file micromachines-09-00364-s001.pdf]

Supplementary Materials: Poly(ionic liquid)s based brush type nanomotor

Experiment section

Characterization Methods

Dynamic light scattering (DLS) measurements were performed on a Malvern Instrument Zetasizer Nano-S (ZEN 1600), equipped with a He-Ne laser (633 nm, 4 mW) and an Avalanche photodiodedetector at an angle of 173°. The DLS data were processed and analyzed with Dispersion Technology Software (Malvern Instruments Ltd., Malvern, UK). Temperature rising rate was set as 1 °C, and waiting time is 10 min for every degree.

Transmission electron microscopy (TEM) was performed JEOL TEM 1010 microscope with an acceleration voltage of 60 kV equipped with a charge-coupled device (CCD) camera. The TEM samples were prepared by placing one drop of the diluted dispersion of PIL nanoparticles on a 200 mesh carboncoated copper grid and left in air to dry.

Cryogenic transmission electron microscopy (cryo-TEM). For plunge freezing, PtNP@NP-*g*-PNIPAM were taken directly at room temperature from the hydrated mixture for processing without further centrifugation. A 3 µl droplet of various mixture samples was applied to a glow-discharge R2/2 quantifoil copper grid (Quantifoil Micro Tools, Jena, Germany) mounted in an environmentally controlled chamber at 100% humidity, blotted and frozen in vitreous ice by plunging into liquid ethane using the Vitrobot (FEI, Eindhoven, The Netherlands). Grids were transferred to a Gatan model 914 cryoholder (Gatan, Pleasanton, CA, USA) under liquid nitrogen and inserted into a Jeol 2100 transmission electron microscope (Jeol, Tokyo, Japan) operating at 200 kV. The vitreous state of the preparation was confirmed by electron diffraction. Low-dose images, with exposures between 20 and 30 electrons per Å2 and underfocus values of 5 µm were recorded with a 4096 × 4096 pixel CCD camera (Gatan) at 40 (thin space (1/6-em)) 000 × magnification.

Proton nuclear magnetic resonance (^{1}H-NMR) spectrum were recorded at ambient temperature using an ARIAN-400 spectrometer operating at 400.1 MHz in CDCl3 solutions. ^{1}H-NMR chemical shifts are given in ppm with respect to tetramethylsilane (TMS, δ 0.00 ppm) as internal standard.

FT-IR spectra were collected with a Varian 1000 FT-IR (scimitar series) spectrometer, equipped with an attenuated total reflection (ATR) setup.

Procedure to Measure Nanomotors by Nanosight

To investigate the motion of the brush nanomotors, nanoparticle tracking analysis (NTA) was carried out with a nanosight LM10 at a magnification of 20 ×. Nanoparticle tracking analysis combines the laser light scattering technique with a CCD camera and is capable of tracking particles of 30 to 1000 nm size moving under Brownian or non-Brownian motion in real-time. The Stokes-Einstein equation can be used to correlate the tracking coordinates obtained from the movement of the particles with their size. The technique is complementary to dynamic light scattering (DLS), however, it provides individual particleby-particle analysis rather than an ensemble size distribution information. Since the method provides additional visualization of the particles we used this technique, in this experiment, to study in detail the effect of the fuel concentration on the movement of the Pt@PNIPAM-PIL brush nanomotors for concentrations ranging from 0.25 and 0.50 vol %.

Freshly prepared hydrogen peroxide was added to 1 mL Pt@PNIPAM-PIL brush nanomotors solution to reach a concentration of 0.25 and 0.50 H_2O_2 vol % before injection into the sample cell. By analyzing the video, x, y coordinates/positions of each particle were determined as a function of time intervals. Mean square displacements obtained by averaging over 30 particles from the major size distribution observed with Nanosight were plotted versus the time intervals. The average velocity

(v) of the nanomotors at these concentrations were extracted from the parabolic fit of the MSD ($< r^2 >$) dependency in time (t) according to the equation $< r^2 > = 4D\Delta t + (v\Delta t)^2$.

Directionality and center of mass are calculated by a software named Chemotaxis and Migration Tool 2.0 from ibidi company.

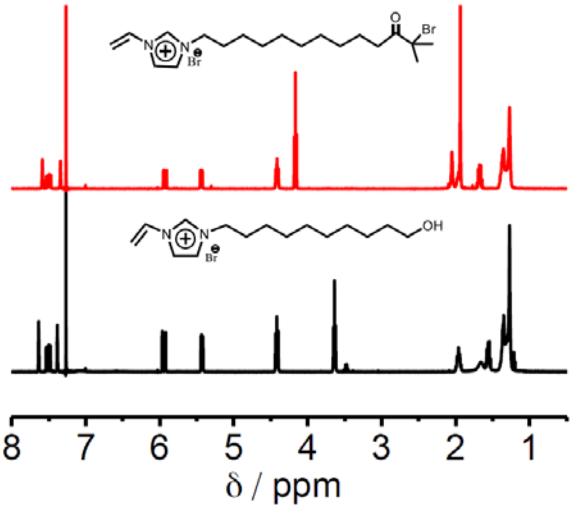

Figure S1. ^{1}H-NMR spectra of 3-(11-hydroxyundecanyl)-1-vinylimidzalium bromide before (**black line**) and after (**red line**) esterification reaction with α-bromoisobutyryl bromide.

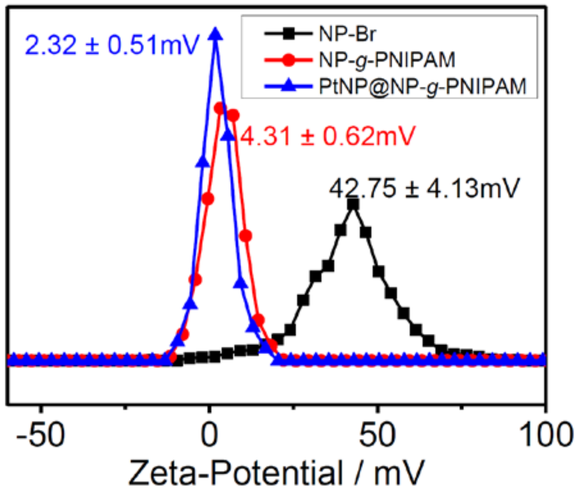

Figure S2. Zeta-Potential values of NP-Br, NP-*g*-PNIPAM and PtNP@NP-*g*-PNIPAM.

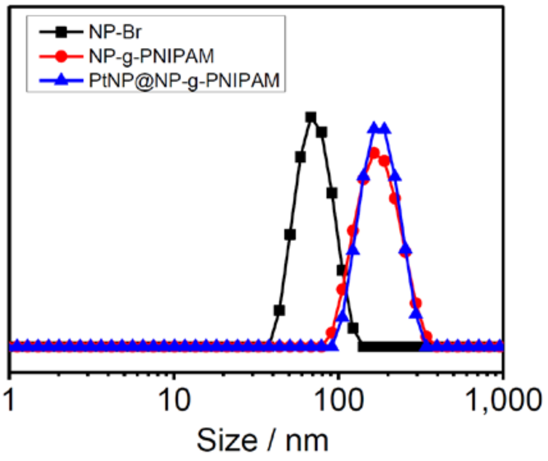

Figure S3. Size of NP-Br, NP-*g*-PNIPAM and PtNP@NP-*g*-PNIPAM measured by DLS.
